# Draft Genome Sequences of Six Strains of *Streptococcus pneumoniae* from Serotypes 5, 6A, 6B, 18C, 19A, and 23F

**DOI:** 10.1128/genomeA.00125-17

**Published:** 2017-04-06

**Authors:** Hedvig E. Jakobsson, Francisco Salvà-Serra, Kaisa Thorell, Roger Karlsson, Lucia Gonzales-Silès, Fredrik Boulund, Lars Engstrand, Erik Kristiansson, Edward R. B. Moore

**Affiliations:** a Department of Infectious Diseases, Sahlgrenska Academy, University of Gothenburg, Gothenburg, Sweden; b Centre for Antibiotic Resistance Research (CARe) at University of Gothenburg, Gothenburg, Sweden; c Microbiology, Department of Biology, University of the Balearic Islands, Palma de Mallorca, Spain; d Department of Microbiology, Tumor and Cell Biology, Karolinska Institutet, Stockholm, Sweden; e Nanoxis Consulting AB, Gothenburg, Sweden; f Department of Mathematical Sciences, Chalmers University of Technology, Gothenburg, Sweden; g Culture Collection University of Gothenburg (CCUG), Gothenburg, Sweden

## Abstract

*Streptococcus pneumoniae* is a pathogenic bacterium found most commonly in the respiratory tract of humans and is a common cause of pneumonia and bacterial meningitis. Here, we report the draft genome sequences of six *S. pneumoniae* strains: CCUG 1350, CCUG 7206, CCUG 11780, CCUG 33774, CCUG 35180, and CCUG 35272.

## GENOME ANNOUNCEMENT

*Streptococcus pneumoniae* (pneumococcus) is an alpha-hemolytic clinically relevant bacterium that causes infections in humans worldwide (1, 2). Although *S. pneumoniae* is observed among the normal nasopharyngeal microbiota in children and resides asymptomatically in most healthy carriers, it is also the most common cause of bacterial meningitis in adults and a common cause of pneumonia and mortality in the elderly (1, 2). There are 97 different serotypes of capsulated pneumococci (3), identified by variations in the polysaccharide capsule. Current 23-valent and 13-valent pneumococcal vaccines target different capsular serotypes (1, 2). In this project, the genome sequences of six *S. pneumoniae* strains, CCUG 1350, CCUG 7206, CCUG 11780, CCUG 33774, CCUG 35180, and CCUG 35272, isolated from different clinical samples in blood, cerebrospinal fluid, and the nasopharynx, have been determined and analyzed. The strains were cultivated and DNA extracted as previously described (4). DNA was sequenced with an Illumina MiSeq instrument (SciLifeLab, Stockholm, Sweden), generating paired-end reads of 300 bp. The data are presented in Table 1. Sequence reads were trimmed and assembled *de novo* with CLC Genomics Workbench version 8 (CLC bio, Aarhus, Denmark). Assembly quality was assessed using Quast version 3.1 (5). Following trimming, the average length of reads was 245 bp. The genome sequences were annotated using the NCBI Prokaryotic Genome Annotation Pipeline (PGAP) (6). The statistics for each draft genome sequence are summarized in Table 1. Comparative analyses of the genome sequences, by average nucleotide identity based on BLAST (ANIb) (7), using JSpecies version 1.2.1 (8), with the genome sequences of the *S. pneumoniae* NCTC 7465 type strain resulted in ANIb values higher than 98%. Comparisons to the type strains of the other species of the Mitis group resulted in ANIb values ranging from 73% to 94%. The analysis of the genome sequences for the presence of clustered regularly interspaced short palindromic repeat (CRISPR)–CRISPR-associated (Cas) systems (9) with CRISPRFinder (10) confirmed the presence of a CRISPR-Cas region in strain CCUG 7206 and one region in strain CCUG 35272 formed by one array of spacer repeats that contained five spacers in CCUG 7206 and three spacers in CCUG 35272. For strains CCUG 1350, CCUG 7206, CCUG 11780, CCUG 33774, and CCUG 35180, no antibiotic resistance genes were found when the genomes were analyzed using ResFinder 2.1 (selected % identification [ID] threshold, 90%). Antibiotic resistance genes were found in strain CCUG 35272: *cat* for chloramphenicol resistance and *tet*(M) for tetracycline resistance, as predicted by phenotype.

**TABLE 1:** Assembly statistics for the six *Streptococcus pneumoniae* draft genome sequences

Strain	Serotype	Accession no.	Coverage (×)	No. of contigs	G+C content (%)	*N*_50_ (kbp)	Genome size (bp)	No. of CDSs^a^
CCUG 7206	18C	LWCD00000000	239	172	39.7	110.5	2,087,379	2,207
CCUG 1350	6B	LQQG00000000	75	91	39.6	83.4	2,147,337	2,138
CCUG 11780	6A	LQQH00000000	82	78	39.5	85.9	2,171,058	2,178
CCUG 33774	5	LQQJ00000000	68	55	39.6	98.2	2,031,618	1,972
CCUG 35180	19A	LQQK00000000	69	75	39.6	84.7	2,087624	2,032
CCUG 35272	23F	LQQI00000000	72	72	39.4	87.6	2,141,195	2,108

a CDSs, coding sequences.

### Accession number(s).

This whole-genome shotgun project has been deposited in DDBJ/ENA/GenBank under the accession numbers listed in Table 1. The versions described in this paper are the first versions, LQQJ01000000, LQQG01000000, LQQH01000000, LQQI01000000, LWCD01000000, and LQQK01000000, for strains CCUG 1350, CCUG 7206, CCUG 11780, CCUG 33774, CCUG 35180, and CCUG 35272, respectively.
